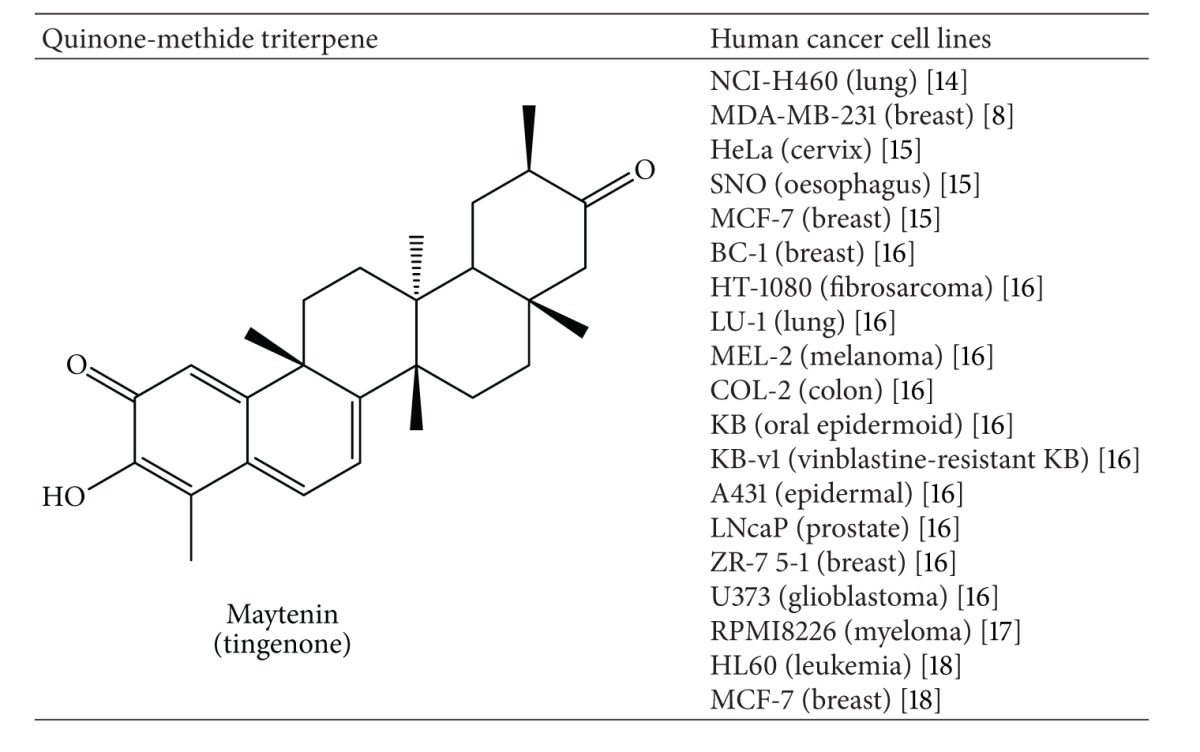# Production of the Quinone-Methide Triterpene Maytenin by *In Vitro* Adventitious Roots of *Peritassa campestris* (Cambess.) A.C.Sm. (Celastraceae) and Rapid Detection and Identification by APCI-IT-MS/MS

**DOI:** 10.1155/2013/485837

**Published:** 2013-09-25

**Authors:** Tiago Antunes Paz, Vânia A. F. F. M. dos Santos, Marielle Cascaes Inácio, Edieidia Souza Pina, Ana Maria Soares Pereira, Maysa Furlan

**Affiliations:** ^1^Núcleo de Bioensaios, Biossíntese e Ecofisiologia de Produtos Naturais, Departamento de Química Orgânica, Instituto de Química, Universidade Estadual Paulista, 14801-970 Araraquara, SP, Brazil; ^2^Departamento de Produção Vegetal, Faculdade de Ciências Agronômicas, Universidade Estadual Paulista, 18610-307 Botucatu, SP, Brazil; ^3^Departamento de Biotecnologia Vegetal, Universidade de Ribeirão Preto, 14096-900, Ribeirão Preto, SP, Brazil

## Abstract

Establishment of adventitious root cultures of *Peritassa campestris* (Celastraceae) was achieved from seed cotyledons cultured in semisolid Woody Plant Medium (WPM) supplemented with 2% sucrose, 0.01% PVP, and 4.0 mg L^−1^ IBA. Culture period on accumulation of biomass and quinone-methide triterpene maytenin in adventitious root were investigated. The accumulation of maytenin in these roots was compared with its accumulation in the roots of seedlings grown in a greenhouse (one year old). A rapid detection and identification of maytenin by direct injection into an atmospheric-pressure chemical ionization ion trap tandem mass spectrometer (APCI-IT-MS/MS) were performed without prior chromatographic separation. *In vitro*, the greatest accumulation of biomass occurred within 60 days of culture. The highest level of maytenin—972.11 **μ**g*·*g^−1^ dry weight—was detected at seven days of cultivation; this value was 5.55-fold higher than that found in the roots of seedlings grown in a greenhouse.

## 1. Introduction

Quinone-methide triterpenes (QMTs) are secondary metabolites that occur only in plants of the Celastraceae family. Therefore, QMTs are considered chemotaxonomic indicators of this family [1, 2]. These terpenes display a wide range of biological activities such as anti-inflammatory [3], antioxidant [4, 5], antifungal [6], antitrypanosomal [7], antimicrobial [8], and mainly antitumor action. Recently, attention has been focused on the QMT maytenin, because it is highly toxic to a panel of cancer cell lines (Table 1). 

Despite the pharmacological potential of maytenin, no reports of their chemical synthesis exist. This is probably because they are structurally complex and contain several stereogenic centers. Hence, extraction from plants may be a feasible strategy to obtain maytenin.

*Peritassa campestris* (Sin. *Salacia campestris*, Celastraceae) is a fruiting shrub native of the Brazilian savanna biome, known as Cerrado; this plant is a source of many QMTs [2, 9]. However, the scale-up production of QMTs from native or cultivated species is not viable: environmental factors can alter the biosynthesis of secondary metabolites, *P. Campestris* grows slowly, and QMTs accumulate only in the roots. Biotechnological techniques such as *in vitro* adventitious roots culture can be considered as alternative to produce these metabolites. Indeed, researchers have employed the adventitious roots of many plant species to obtain secondary metabolites [10–13].

In this context, this study aimed (1) to establish a protocol for cultivation of *P. campestris* adventitious root; (2) to assay atmospheric-pressure chemical ionization ion trap tandem mass spectrometer (APCI-IT-MS/MS), without prior chromatographic separation, as a rapid and sensitive method to screen maytenin in root extracts; and (3) to gather quantitative information about the growth and biosynthesis of QMT in the adventitious root cultures. 

## 2. Materials and Methods

### 2.1. Plant Material, Seedlings, and Adventitious Root Culture

Adult plants and seeds of *P. campestris* were collected from the Brazilian tropical savanna (Cerrado) in Catalão, GO, Brazil; the specimen was identified by Dr. Julio Antonio Lombardi (Instituto de Biociências, UNESP, Rio Claro, SP). A voucher specimen (1415) was deposited at the Herbarium of Medicinal Plants of the University of RibeirãoPreto (HPM-UNAERP, Ribeirão Preto, SP). Seedlings were grown from seeds of *P. campestris* using native soil in a greenhouse for one year.

**Figure 1:**
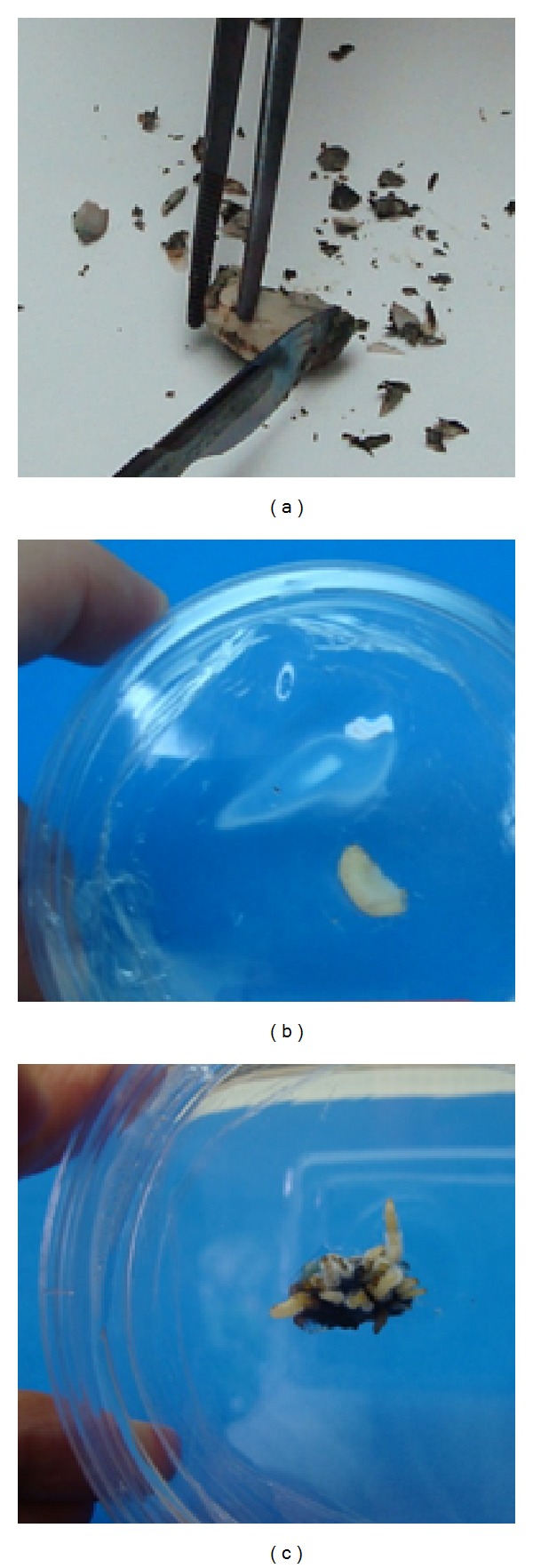
Initialization of *in vitro* adventitious roots of *Peritassa campestris*. (a) The seed coats being removed and the cotyledons being cut into explants; (b) seed cotyledon explants placed in WPM medium supplemented with 2% (w/v) sucrose, 0.01% (w/v) PVP, and 4.0 mg·L^−1^ IBA; (c) adventitious roots after incubation in the dark for four weeks.

To initiate the adventitious root culture, *P. campestris* seeds were sterilized with a solution containing thiophanate-methyl (1%, w/v), captan (1%, w/v), kanamycin, ampicillin, chloramphenicol, and cefotaxime (all 0.1%, w/v) under shaking at 80 rpm for 24 h, followed by addition of 0.5% (w/v) calcium hypochlorite for 15 min. Seeds were then rinsed three times with sterile distilled water, and their coats were removed (Figure 1(a)); the cotyledons were cut into explants (4/seed of 3–5 mm^2^) and placed in Woody Plant Medium (WPM) [19] and MS [20] supplemented with, respectively, 2% and 3% (w/v) sucrose: 0.01% (w/v) polyvinylpyrrolidone (PVP), and the auxins indole-3-butyric acid (IBA), 2,4-dichlorophenoxyacetic acid (2,4-D), or 1-naphthaleneacetic acid (NAA) in different concentrations (1.0, 2.0, 3.0, 4.0, or 6.0 mg·L^−1^). All media were adjusted to pH 6.0 and solidified with 0.2% (w/v) Phytagel prior to autoclaving at 121°C for 15 min. The cultures were incubated at 25 ± 2°C, in the dark; adventitious roots appeared after four weeks. These roots were maintained by subculturing in fresh WPM supplemented with 2% sucrose, 0.01% PVP, and 4.0 mg·L^−1^ IBA every four weeks.

### 2.2. Determination of the Root Weight

One-year-old adventitious roots (fresh weight *≈* 1 g) were transferred to flasks containing 15 mL of fresh medium (WPM supplemented with 2% sucrose, 0.01% PVP, and 4.0 mg·L^−1^ IBA) and maintained under the same culture conditions described above. At the end of 0, 7, 15, 30, 45, 60, 75, 90, and 105 days, the roots were removed from the culture medium, and their fresh weight (FW) was recorded. The dry weight (DW) of the adventitious root was accurately measured after roots for 24 h in an oven at 40°C. The growth curve was plotted according to the DW of the biomass of the roots. The content of the bioactive compound maytenin was also determined. 

### 2.3. Preparation of Extracts

**Figure 2:**
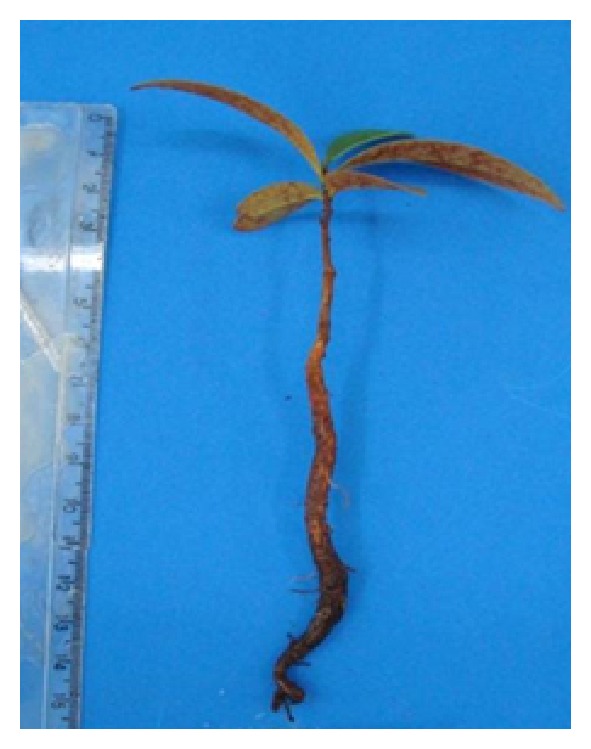
Seedling of *Peritassa campestris* grown in a greenhouse (one year old).

The roots of seedlings grown in a greenhouse (one year old) (Figure 2) and the adventitious roots were dried and ground into a fine powder. They were then sonicated with dichloromethane (1 g DW/10 mL solvent) 3 times for 20 min, at room temperature. Next, the samples were filtered on filter paper and evaporated to dryness.

### 2.4. Detection and Identification of Maytenin by Mass Spectrometry

To detect and identify QMTs in the extracts of the roots, mass spectrometry based on the detection of the protonated molecular ion peaks [M + H]^+^ and their well-known fragmentation patterns [21–24] was conducted. Analyses were performed using direct flow injection atmospheric-pressure chemical ionization ion trap tandem mass spectrometry (APCI-IT-MS/MS).

The root extracts were dissolved in methanol (1.0 *μ*g DW mL^−1^) and filtered through a 0.22 *μ*m membrane of a nylon filter. The solution was directly introduced into the APCI source by flow injection at 5 *μ*L min^−1^, using a syringe pump. The MS and MS/MS analyses were accomplished on a Thermo Scientific LCQ Fleet ion trap spectrometer equipped with an APCI interface operating in the positive ion mode. The stainless steel capillary temperature was set to 200°C; the pressure of the nebulizing gas (nitrogen) was 88 psi.

### 2.5. Preparation of Samples for HPLC Analysis

The root extracts were dissolved in methanol/water (95 : 5, v/v) (2.5 mg DW mL^−1^). Next, a 1 mL aliquot of the resulting solution was submitted to solid-phase extraction (SPE) SPE cartridges (Sampliq Agilent) (3 mL) containing 500 mg of C_18_ silica gel (particle size of 40 *μ*m) preconditioned with methanol and methanol/water (95 : 05 v/v). Cartridges were eluted with 3 mL of methanol/water (95 : 05 v/v). The interfering compounds were retained by the C_18_ silica gel, whilst the eluate was transferred to a 5 mL volumetric flask, adjusted to the mark with the same 95% methanol. The resulting solution was filtered through a 0.22 *μ*m membrane of a nylon filter, and an aliquot (20 *μ*L) was assayed by HPLC.

### 2.6. Quantitative HPLC Analysis of Maytenin

The content of maytenin in the root extracts was determined using the method described by Corsino at al. [25] with a few modifications. The samples were analyzed on an HPLC system from Shimadzu (Kyoto, Japan), namely, an LC-10-AVP instrument equipped with an autoinjector SIL-10AF, a photodiode array detector SPD-M20A, and a Phenomenex (Torrance, CA, USA) Luna C_18_ column (250 × 4.6 mm i.d.; particle size of 5 *μ*m). The mobile phase was methanol/water/formic acid (80 : 20 : 0.1, v/v/v); elution was carried out in the isocratic mode for 20 min, at a flow rate of 1 mL min^−1^. The injection volume was 20 *μ*L for the samples and the chemical standard; detection was conducted at a wavelength of 420 nm. Maytenin (HPLC purity > 98%), isolated and identified by NMR and MS experiments in our laboratory from the root barks of *P. campestris*, was employed as chemical standard. Measurements were integrated by comparison with an external standard calibration curve. Limits of detection and quantification were, respectively, 41,00 × 10^−3^ and 124,0 × 10^−3^ *μ*g·mL^−1^.

## 3. Results and Discussion

### 3.1. Induction and Maintenance of Adventitious Roots

Plant cell suspension cultures are the most popular strategy among the *in vitro* plant culture techniques—it is simple, provides homogenous and fast growing material, and is easy to scale up. However, the biosynthetic machinery necessary to generate the various target metabolites may not be available or may afford only small amounts of the desired compounds. In such an event, a plant organ culture might be a preferable system [26].

**Figure 3:**
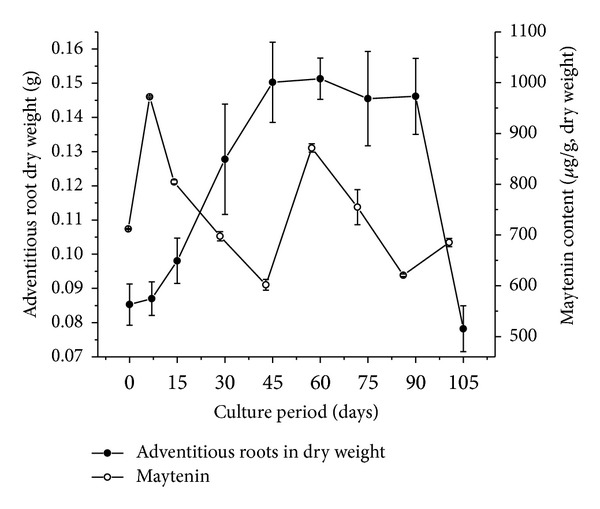
Adventitious root growth of *P. campestris* in dry weight and production of maytenin. The roots were cultivated for 105 days in WPM medium supplemented with 2% (w/v) sucrose, 0.01% (w/v) PVP, and 4.0 mg·L^−1^ IBA under dark conditions.

We verified initial formation of adventitious roots of *P. campestris *after four weeks of inoculation in WPM supplemented with 2% (w/v) sucrose, 0.01% (w/v) PVP, and 4.0 mg·L^−1^ IBA (Figure 1(c)). The roots present typical growth: a lag phase from 0 to 7 days, an exponential phase from 7 to 45 days, a stationary phase from 45 to 90 days, and a declining phase thereafter (Figure 3). After 45–60 days of cultivation, the biomass peaks and is approximately twice the dry weight (DW) of the initial inoculum. MS and HPLC-DAD analyses confirm that the *in vitro *adventitious roots produce maytenin at various concentrations during all growth phases (Figure 3).

The increased production of maytenin during the first seven days of culture does not correspond to accumulation of adventitious root biomass. The content of maytenin (972.11 *μ*g·g^−1^ DW) peaks at seven days. A similar growth pattern was observed for a culture of *Maytenus ilicifolia* (Celastraceae) callus—production of this same metabolite peaked in the log phase (16 days of cultivation, 44.0 *μ*g·g^−1^ DW) [27]. During the growth phase, between 15 and 45 days, the DW of the root cultures increases by 52.28%; in contrast, the production of maytenin decreases (from 804.60 to 601.98 *μ*g·g^−1^ DW). Buffa-Filho et al. [27] also verified that cultures of *M. ilicifolia *callus produce lower amounts of this QMT in the same growth phase. These results were expected since plant cells usually produce primary metabolites at the expense of secondary metabolites during the linear growth phase.

Between 45 and 60 days of cultivation, the concentration of maytenin increases (from 601.98 to 871.26 *μ*g·g^−1^ DW); in the subsequent 30 days, it decreases (620.83 *μ*g·g^−1^ DW), whereas the adventitious root remains in the stationary phase. After 90 days of cultivation, the adventitious roots begin to decline, and the concentration of maytenin increases slightly.

Together, these results indicate that the scale production of maytenin from these roots is maximized using the following protocol: the adventitious roots of *P. campestris *grow for 60 days in the conditions described here, when the largest amount of biomass is achieved and maytenin concentration attains the maximum value. By this time, the roots should be removed and transferred to fresh culture medium for additional seven days. This should generate maximum concentration of the target metabolite from a large amount of biomass. 

### 3.2. Comparing the Production of Maytenin by Roots of Seedlings and Adventitious Roots of *P. campestris*

Analyses reveal that both, the roots from seedlings of* P. campestris *grown in a greenhouse (one year old) and the adventitious roots biosynthesize maytenin, however in different amounts. After seven days of *in vitro* culture (peak of production), the adventitious roots produce 5.55 times more maytenin than the roots of seedlings (972.11 and 175.27 *μ*g·g^−1^ DW, resp.).

Higher production of secondary metabolites in tissue cultures as compared with *in vivo* plants is not a rule. The concentration of any metabolite may be higher or lower, depending on factors such as light, culture medium, and growth regulators, among others [28–30]. Concerning the species *P. campestris*, the greater accumulation of QMTs by the *in vitro *adventitious root may be associated with the presence of IBA. Accordingly, [31] auxins activate genes that regulate cytochrome P450 enzymes, which are involved in the biosynthesis of terpenoids.

### 3.3. Detection and Identification of QMT Maytenin by APCI-IT-MS/MS

A variety of screening methods exist to detect and identify known and unknown metabolites in plant materials [32]. However, for* in vitro *lab-scale plant cultures, the most suitable method should be fast and highly sensitive, given the limited quantities of biomass. MS is a method that meets all these requirements, even in the absence of reference standards [32].

**Figure 4:**
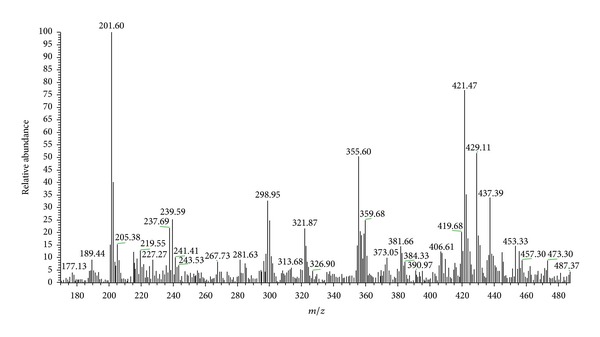
Full APCI-IT-MS mass spectrum of the extract from the *in vitro *adventitious roots of *Peritassa campestris *obtained in the positive ion mode, normal mass scan, in the *m/z* range of 170–490.

**Figure 5:**
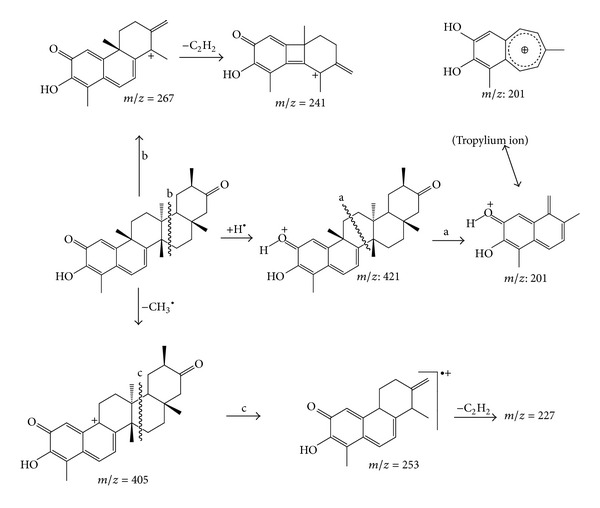
Main fragmentation pathways observed in the positive ion mass spectrum of maytenin (adapted from [21]).

The full mass spectrum of the adventitious root extracts of *P. campestris *(Figure 4) is a complex spectrum with signals of ions from the different compounds present in the sample. However, the base peak at *m/z* 201 in the spectrum suggests the presence of QMTs in this extract; indeed, this peak refers to the formation of a fragment of a tropylium structure (Figure 5), which is typical of these compounds [21, 24]. The ion at *m/z* 421 corresponds to the protonated maytenin [M + H]^+^.

**Figure 6:**
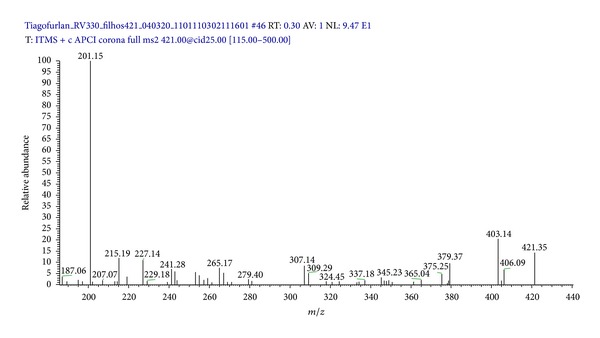
Spectrum of the [M + H]^+^ ion at *m/z* 421 of maytenin in the extract from *in vitro *adventitious roots of *Peritassa campestris*.

The MS/MS spectrum of *m/z* 421 (Figure 6) reveals the presence of the base peak *m/z* 201 and of the mass fragments at *m/z* 227, 241, 253, and 267, characteristic of the quinone methides [21, 22]. Therefore, the ion at *m/z* 421 is the protonated maytenin [M + H]^+^. Figure 5 shows the main fragmentation pathways of maytenin.

## 4. Conclusions

An efficient protocol to grow adventitious roots of *P. campestris in vitro* was successfully developed using cotyledons of seeds placed in semisolid WPM supplemented with 2% (w/v) sucrose, 0.01% (w/v) PVP, and 4.0 mg·L^−1^ IBA under dark conditions. The productivity of maytenin in the *in vitro* roots is higher comparing with the *in vivo* production by the roots of seedlings. These results indicate that the adventitious roots cultures of *P. campestris* are a promising source of maytenin and a base for scaling-up adaptations in view of its future production in bioreactors.

APCI-IT-MS/MS analyses using direct flow injection of the root extracts of *P. campestris* are a fast, simple, and efficient strategy to screen the QMT maytenin.

## Supplementary Material

MS, 1H, and 13C NMR (1D and 2D) spectra of maytenin.

## Figures and Tables

**Table 1:** *In vitro* effects of maytenin on cancer cell lines.