# Supplementary material for: Production of the Quinone-Methide Triterpene Maytenin by In Vitro Adventitious Roots of Peritassa campestris (Cambess.) A.C.Sm. (Celastraceae) and Rapid Detection and Identification by APCI-IT-MS/MS
Source: Biomed Res Int. 2013 Sep 25;2013:485837. doi: 10.1155/2013/485837 (PMC3800617; doi:10.1155/2013/485837)
Supplement: Supplementary file 1 — MS, 1H, and 13C NMR (1D and 2D) spectra of maytenin. [file 485837.f1.pdf]

Generic Display Report

Faculdade de Ciencias Farmaceuticas de Ribeirao Preto-USP

Analysis Info

Analysis Name	D:\Data\SILVIA T\TINGENONE_POS.d	Acquisition Date	8/26/2013 4:50:56 PM
Method	Tune_Low_Tomaz_Pos_1300u_NOVO.m	Operator	BDAL@DE
Sample Name	TINGENONE_POS	Instrument	micrOTOF-Q II
Comment			

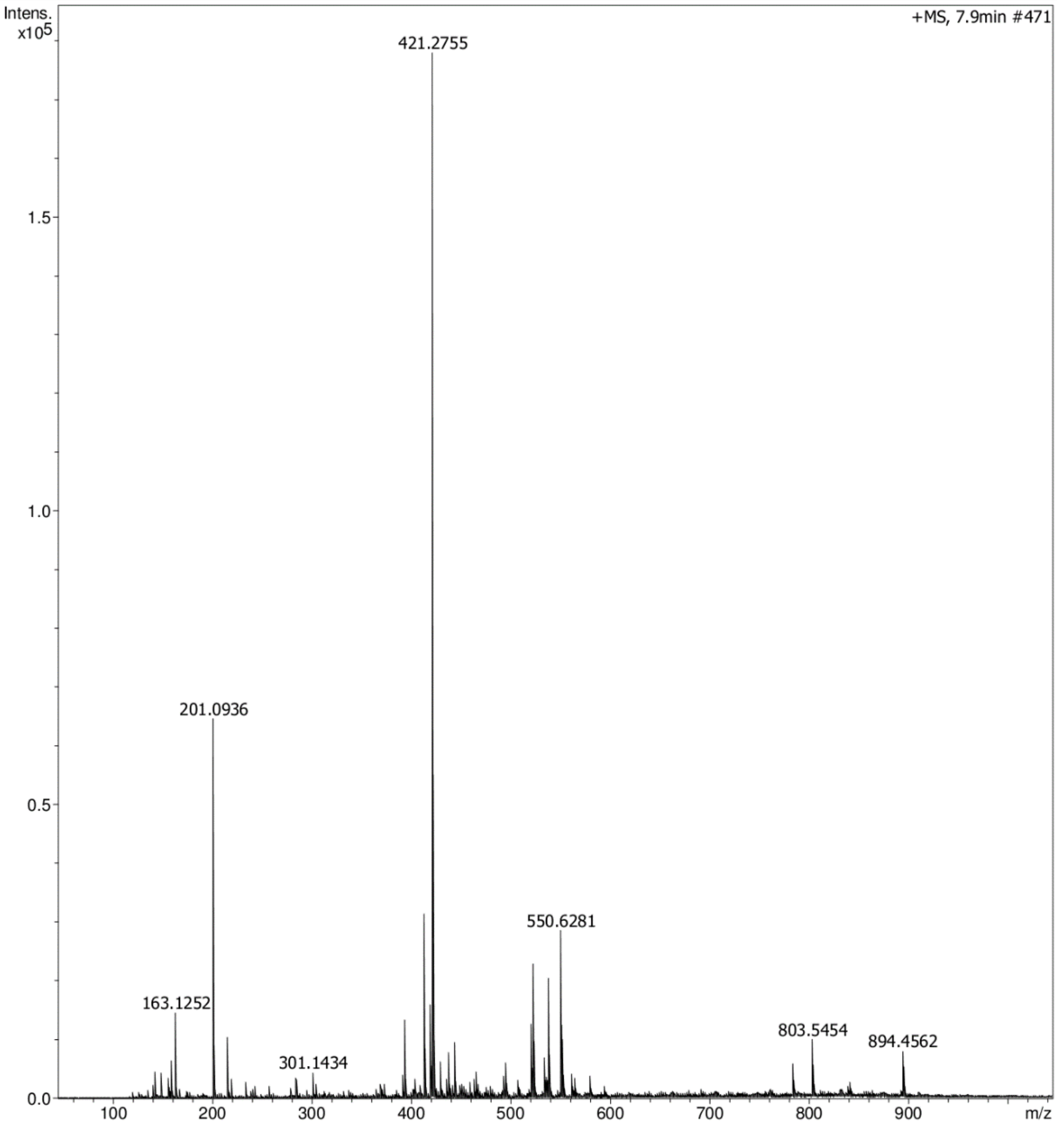

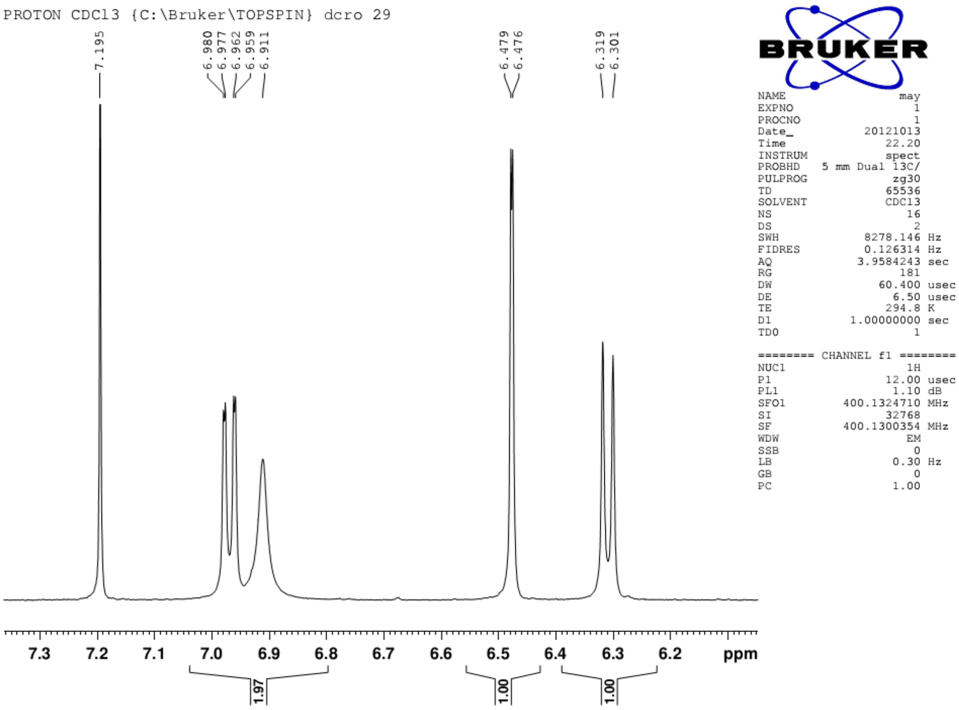
PROTON CDCl3 {C:\Bruker\TOPSPIN} dcro 29
7.195
6.980
6.977
6.962
6.959
6.911
6.479
6.476
6.319
6.301
BRUKER
NAME may
EXPNO 1
PROCNO 1
Date_ 20121013
Time 22.20
INSTRUM spect
PROBHD 5 mm Dual 13C/
PULPROG zg30
TD 65536
SOLVENT CDCl3
NS 16
DS 2
SWH 8278.146 Hz
FIDRES 0.126314 Hz
AQ 3.9584243 sec
RG 181
DW 60.400 usec
DE 6.50 usec
TE 294.8 K
D1 1.00000000 sec
TD0 1
======== CHANNEL f1 ========
NUC1 1H
P1 12.00 usec
PL1 1.10 dB
SFO1 400.1324710 MHz
SI 32768
SF 400.1300354 MHz
WDW EM
SSB 0
LB 0.30 Hz
GB 0
PC 1.00
7.3 7.2 7.1 7.0 6.9 6.8 6.7 6.6 6.5 6.4 6.3 6.2 ppm
1.97
1.00
1.00

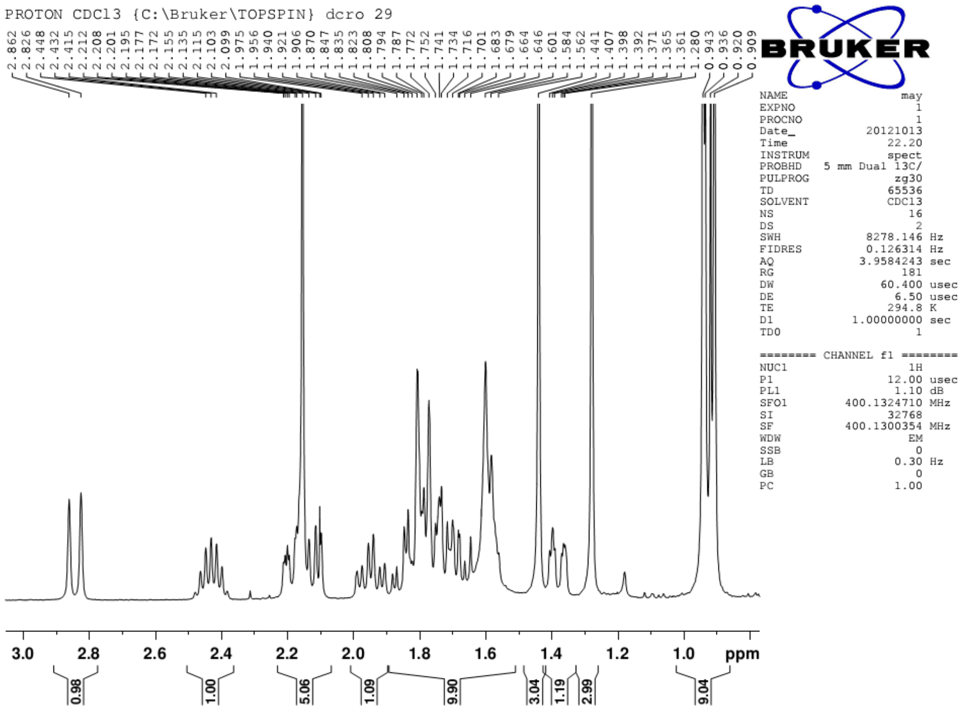
PROTON CDCl3 {C:\Bruker\TOPSPIN} dcro 29
2.862
2.826
2.448
2.432
2.415
2.212
2.208
2.201
2.195
2.177
2.172
2.155
2.135
2.115
2.103
2.099
1.975
1.956
1.940
1.921
1.906
1.870
1.847
1.835
1.823
1.808
1.794
1.787
1.772
1.752
1.741
1.734
1.716
1.701
1.683
1.679
1.664
1.646
1.601
1.584
1.562
1.441
1.407
1.398
1.392
1.371
1.365
1.361
1.280
0.943
0.936
0.920
0.909
BRUKER
NAME may
EXPNO 1
PROCNO 1
Date_ 20121013
Time 22.20
INSTRUM spect
PROBHD 5 mm Dual 13C/
PULPROG zg30
TD 65536
SOLVENT CDCl3
NS 16
DS 2
SWH 8278.146 Hz
FIDRES 0.126314 Hz
AQ 3.9584243 sec
RG 181
DW 60.400 usec
DE 6.50 usec
TE 294.8 K
D1 1.00000000 sec
TD0 1
======== CHANNEL f1 ========
NUC1 1H
P1 12.00 usec
PL1 1.10 dB
SFO1 400.1324710 MHz
SI 32768
SF 400.1300354 MHz
WDW EM
SSB 0
LB 0.30 Hz
GB 0
PC 1.00
3.0 2.8 2.6 2.4 2.2 2.0 1.8 1.6 1.4 1.2 1.0 ppm
0.98
1.00
5.06
1.09
9.90
3.04
1.19
2.99
9.04

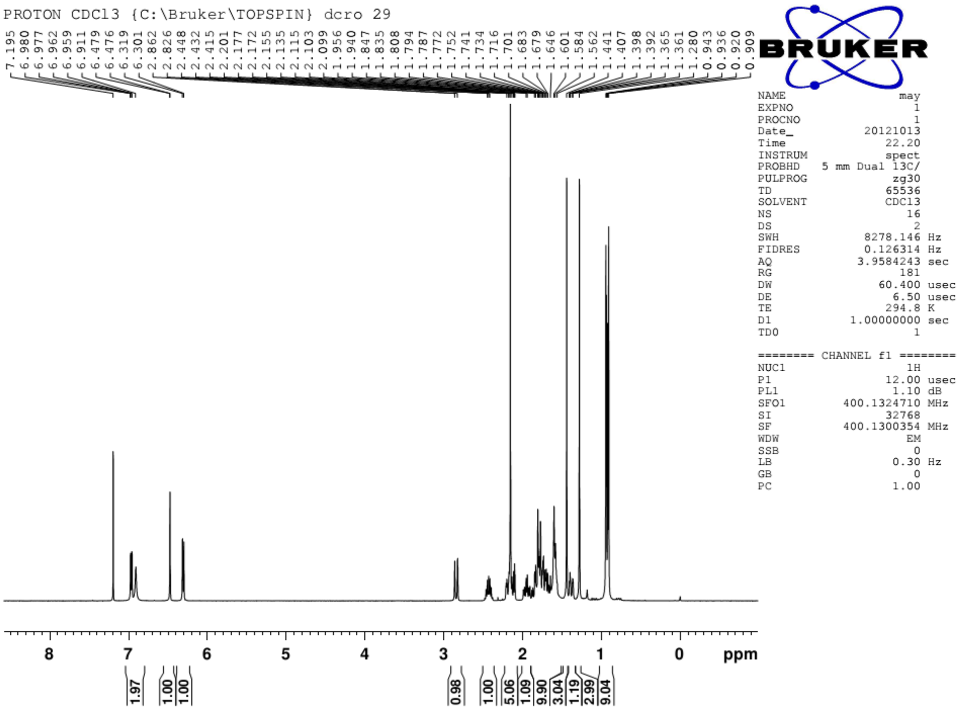
PROTON CDCl3 {C:\Bruker\TOPSPIN} dcro 29
7.195 6.980 6.977 6.962 6.959 6.911 6.479 6.476 6.319 6.301 2.862 2.826 2.448 2.432 2.415 2.201 2.177 2.172 2.155 2.135 2.115 2.103 2.099 1.956 1.940 1.847 1.835 1.808 1.794 1.787 1.772 1.752 1.741 1.734 1.716 1.701 1.683 1.679 1.646 1.601 1.584 1.562 1.441 1.407 1.398 1.392 1.365 1.361 1.280 0.943 0.936 0.920 0.909
BRUKER
NAME may
EXPNO 1
PROCNO 1
Date_ 20121013
Time 22.20
INSTRUM spect
PROBHD 5 mm Dual 13C/
PULPROG zg30
TD 65536
SOLVENT CDCl3
NS 16
DS 2
SWH 8278.146 Hz
FIDRES 0.126314 Hz
AQ 3.9584243 sec
RG 181
DW 60.400 usec
DE 6.50 usec
TE 294.8 K
D1 1.00000000 sec
TD0 1
======== CHANNEL f1 ========
NUC1 1H
P1 12.00 usec
PL1 1.10 dB
SFO1 400.1324710 MHz
SI 32768
SF 400.1300354 MHz
WDW EM
SSB 0
LB 0.30 Hz
GB 0
PC 1.00
8 7 6 5 4 3 2 1 0 ppm
1.97 1.00 1.00 0.98 1.00 5.06 1.09 9.90 3.04 1.19 2.99 9.04

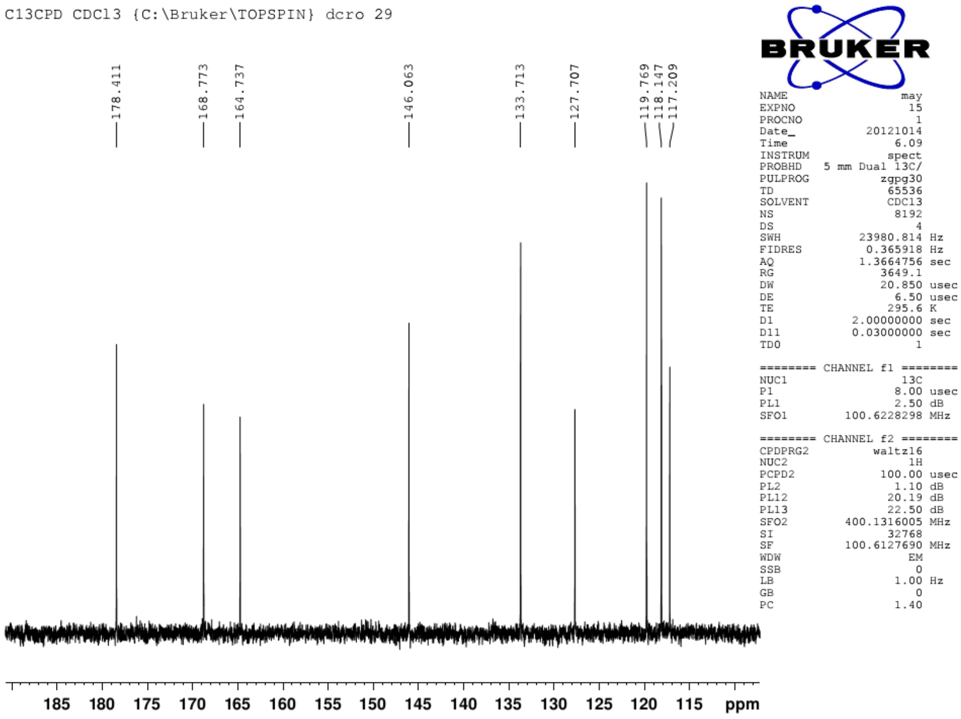
C13CPD CDCl3 {C:\Bruker\TOPSPIN} dcro 29
178.411 168.773 164.737 146.063 133.713 127.707 119.769 118.147 117.209
BRUKER
NAME may
EXPNO 15
PROCNO 1
Date_ 20121014
Time 6.09
INSTRUM spect
PROBHD 5 mm Dual 13C/
PULPROG zgpg30
TD 65536
SOLVENT CDCl3
NS 8192
DS 4
SWH 23980.814 Hz
FIDRES 0.365918 Hz
AQ 1.3664756 sec
RG 3649.1
DW 20.850 usec
DE 6.50 usec
TE 295.6 K
D1 2.00000000 sec
D11 0.03000000 sec
TD0 1
======== CHANNEL f1 ========
NUC1 13C
P1 8.00 usec
PL1 2.50 dB
SFO1 100.6228298 MHz
======== CHANNEL f2 ========
CPDPRG2 waltz16
NUC2 1H
PCPD2 100.00 usec
PL2 1.10 dB
PL12 20.19 dB
PL13 22.50 dB
SFO2 400.1316005 MHz
SI 32768
SF 100.6127690 MHz
WDW EM
SSB 0
LB 1.00 Hz
GB 0
PC 1.40
185 180 175 170 165 160 155 150 145 140 135 130 125 120 115 ppm

C13CPD CDCl3 {C:\Bruker\TOPSPIN} dcro 29

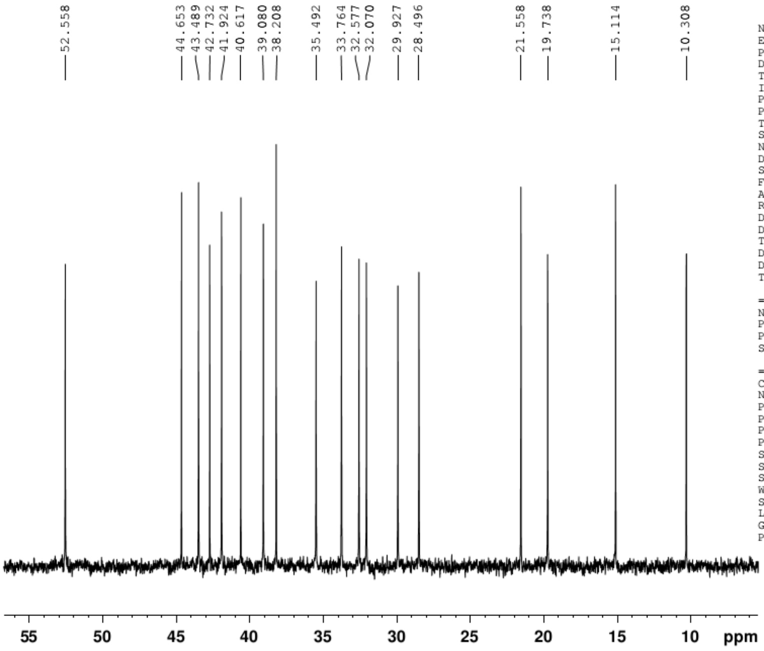

BRUKER

```
NAME              may
EXPNO              15
PROCNO              1
Date_        20121014
Time             6.09
INSTRUM         spect
PROBHD   5 mm Dual 13C/
PULPROG        zgpg30
TD              65536
SOLVENT         CDCl3
NS               8192
DS                  4
SWH         23980.814 Hz
FIDRES       0.365918 Hz
AQ          1.3664756 sec
RG             3649.1
DW             20.850 usec
DE               6.50 usec
TE              295.6 K
D1         2.00000000 sec
D11        0.03000000 sec
TD0                 1

======== CHANNEL f1 ========
NUC1              13C
P1               8.00 usec
PL1              2.50 dB
SFO1      100.6228298 MHz

======== CHANNEL f2 ========
CPDPRG2       waltz16
NUC2               1H
PCPD2          100.00 usec
PL2              1.10 dB
PL12            20.19 dB
PL13            22.50 dB
SFO2      400.1316005 MHz
SI              32768
SF        100.6127690 MHz
WDW                EM
SSB                 0
LB               1.00 Hz
GB                  0
PC               1.40
```

C13CPD CDCl3 {C:\Bruker\TOPSPIN} dcro 29

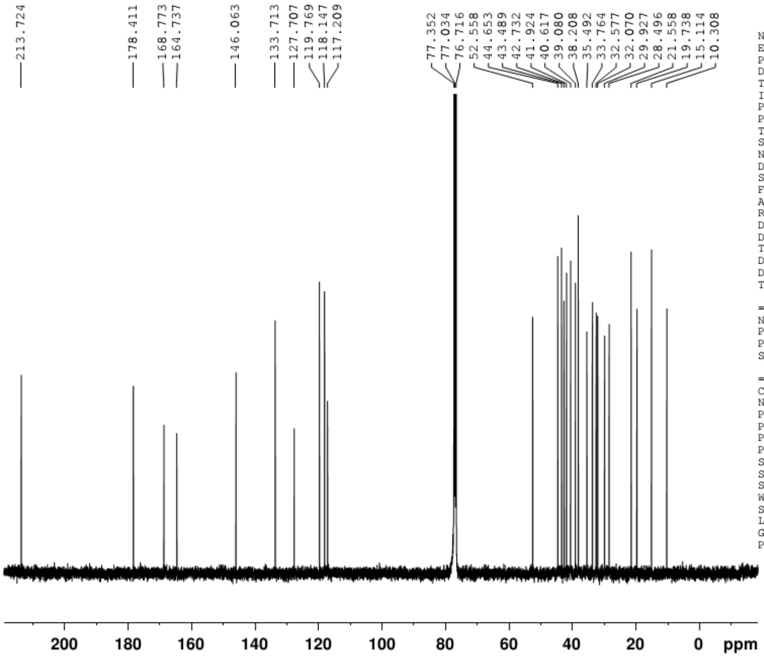

BRUKER

```
NAME              may
EXPNO              15
PROCNO              1
Date_        20121014
Time             6.09
INSTRUM         spect
PROBHD   5 mm Dual 13C/
PULPROG        zgpg30
TD              65536
SOLVENT         CDCl3
NS               8192
DS                  4
SWH         23980.814 Hz
FIDRES       0.365918 Hz
AQ          1.3664756 sec
RG             3649.1
DW             20.850 usec
DE               6.50 usec
TE              295.6 K
D1         2.00000000 sec
D11        0.03000000 sec
TD0                 1

======== CHANNEL f1 ========
NUC1              13C
P1               8.00 usec
PL1              2.50 dB
SFO1      100.6228298 MHz

======== CHANNEL f2 ========
CPDPRG2       waltz16
NUC2               1H
PCPD2          100.00 usec
PL2              1.10 dB
PL12            20.19 dB
PL13            22.50 dB
SFO2      400.1316005 MHz
SI              32768
SF        100.6127690 MHz
WDW                EM
SSB                 0
LB               1.00 Hz
GB                  0
PC               1.40
```

COSYGPSW CDCl3 {C:\Bruker\TOPSPIN} dcro 29

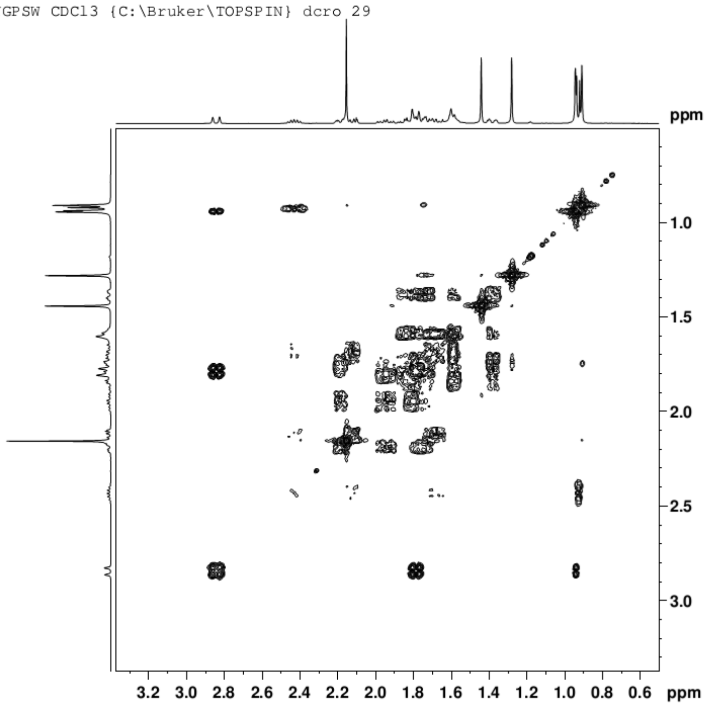

NAME may
EXPNO 300
PROCNO 1
Date_ 20121014
Time 9.06
INSTRUM spect
PROBHD 5 mm Dual 13C/
PULPROG cosygpqf
TD 4096
SOLVENT CDCl3
NS 8
DS 8
SWH 2997.602 Hz
FIDRES 0.731836 Hz
AQ 0.6832628 sec
RG 90.5
DW 166.800 usec
DE 6.50 usec
TE 295.0 K
D0 0.00000300 sec
D1 1.18624604 sec
D13 0.00000400 sec
D16 0.00020000 sec
IN0 0.00033360 sec

======== CHANNEL f1 ========
NUC1 1H
P0 12.00 usec
P1 12.00 usec
PL1 1.10 dB
SFO1 400.1315246 MHz

====== GRADIENT CHANNEL =====
GPNAM1 SINE.100
GPZ1 10.00 %
P16 1000.00 usec
ND0 1
TD 320
SFO1 400.1315 MHz
FIDRES 9.367506 Hz
SW 7.492 ppm
FnMODE QF
SI 1024
SF 400.1300354 MHz
WDW SINE
SSB 0
LB 0.00 Hz
GB 0
PC 1.40
SI 1024
MC2 QF
SF 400.1300354 MHz
WDW SINE
SSB 0
LB 0.00 Hz
GB 0

COSYGPSW CDCl3 {C:\Bruker\TOPSPIN} dcro 29

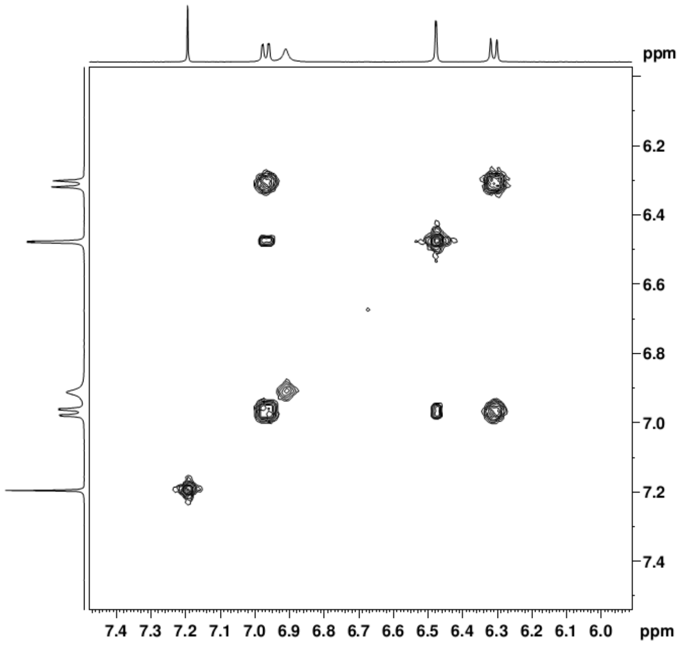

NAME may
EXPNO 300
PROCNO 1
Date_ 20121014
Time 9.06
INSTRUM spect
PROBHD 5 mm Dual 13C/
PULPROG cosygpqf
TD 4096
SOLVENT CDCl3
NS 8
DS 8
SWH 2997.602 Hz
FIDRES 0.731836 Hz
AQ 0.6832628 sec
RG 90.5
DW 166.800 usec
DE 6.50 usec
TE 295.0 K
D0 0.00000300 sec
D1 1.18624604 sec
D13 0.00000400 sec
D16 0.00020000 sec
IN0 0.00033360 sec

======== CHANNEL f1 ========
NUC1 1H
P0 12.00 usec
P1 12.00 usec
PL1 1.10 dB
SFO1 400.1315246 MHz

====== GRADIENT CHANNEL =====
GPNAM1 SINE.100
GPZ1 10.00 %
P16 1000.00 usec
ND0 1
TD 320
SFO1 400.1315 MHz
FIDRES 9.367506 Hz
SW 7.492 ppm
FnMODE QF
SI 1024
SF 400.1300354 MHz
WDW SINE
SSB 0
LB 0.00 Hz
GB 0
PC 1.40
SI 1024
MC2 QF
SF 400.1300354 MHz
WDW SINE
SSB 0
LB 0.00 Hz
GB 0

COSYGPSW CDCl3 {C:\Bruker\TOPSPIN} dcro 29

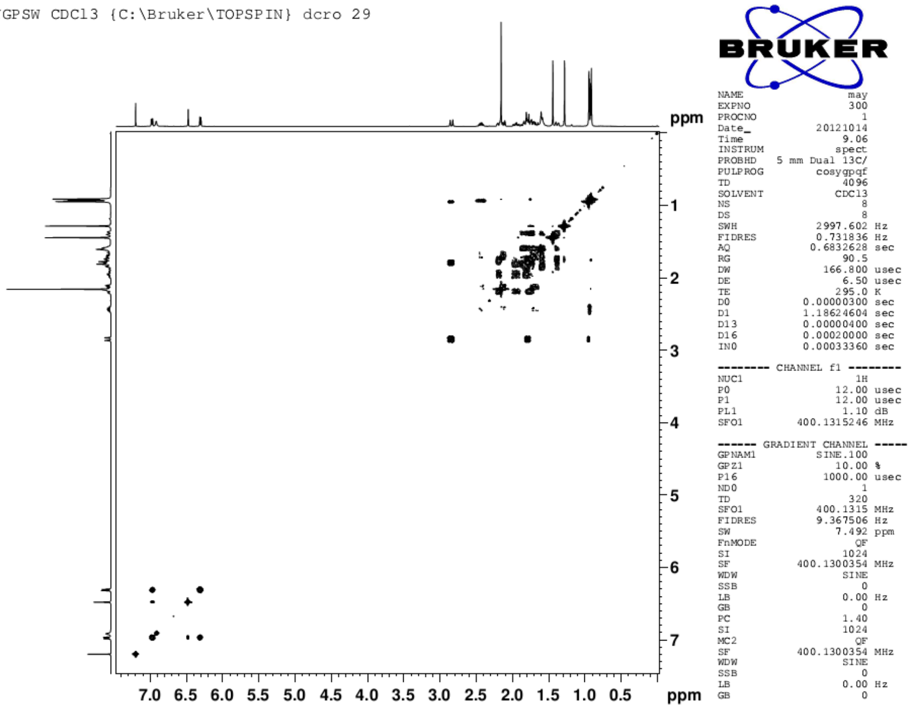

C13DEPT135 CDCl3 {C:\Bruker\TOPSPIN} dcro 29

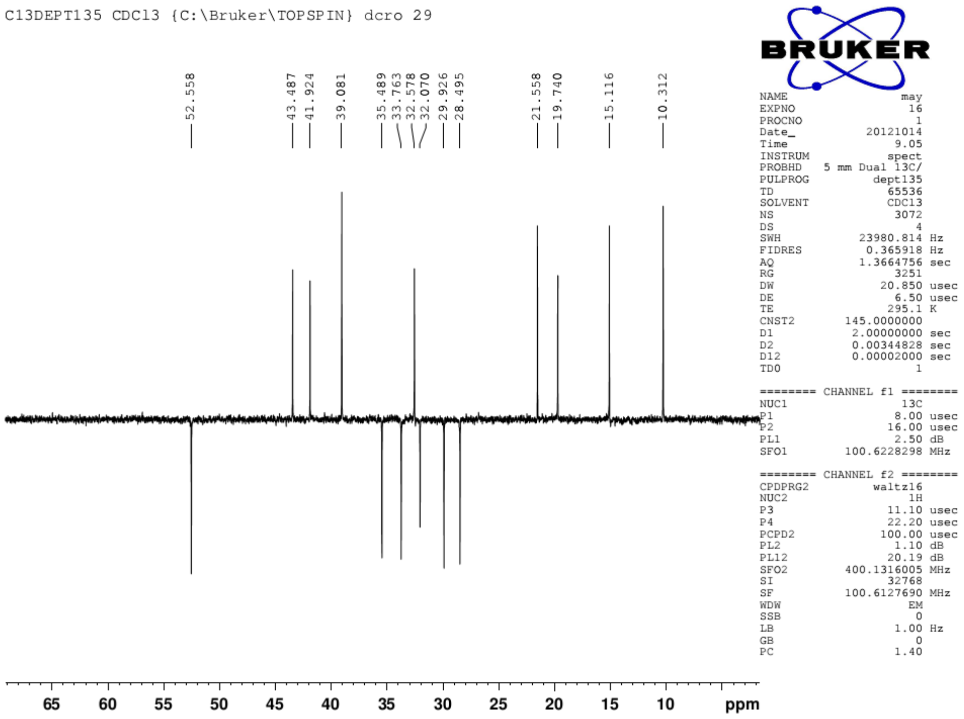

C13DEPT135 CDCl3 {C:\Bruker\TOPSPIN} dcro 29

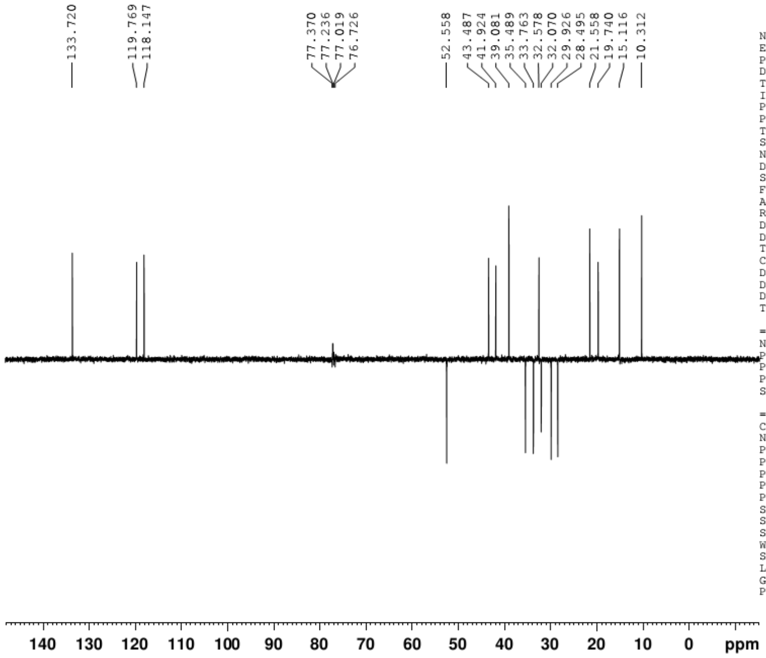

BRUKER

NAME may
EXPNO 16
PROCNO 1
Date_ 20121014
Time 9.05
INSTRUM spect
PROBHD 5 mm Dual 13C/
PULPROG dept135
TD 65536
SOLVENT CDCl3
NS 3072
DS 4
SWH 23980.814 Hz
FIDRES 0.365918 Hz
AQ 1.3664756 sec
RG 3251
DW 20.850 usec
DE 6.50 usec
TE 295.1 K
CNST2 145.0000000
D1 2.00000000 sec
D2 0.00344828 sec
D12 0.00002000 sec
TD0 1

======== CHANNEL f1 ========
NUC1 13C
P1 8.00 usec
P2 16.00 usec
PL1 2.50 dB
SFO1 100.6228298 MHz

======== CHANNEL f2 ========
CPDPRG2 waltz16
NUC2 1H
P3 11.10 usec
P4 22.20 usec
PCPD2 100.00 usec
PL2 1.10 dB
PL12 20.19 dB
SFO2 400.1316005 MHz
SI 32768
SF 100.6127690 MHz
WDW EM
SSB 0
LB 1.00 Hz
GB 0
PC 1.40

HMBCGP CDCl3 {C:\Bruker\TOPSPIN} dcro 29

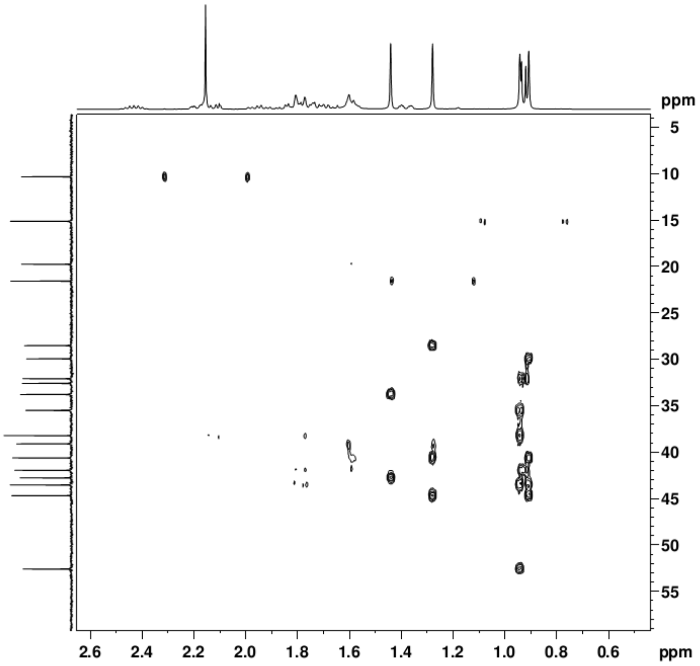

BRUKER

NAME may
EXPNO 500
PROCNO 1
Date_ 20121014
Time 13.14
INSTRUM spect
PROBHD 5 mm Dual 13C/
PULPROG hmbcgplpndqf
TD 4096
SOLVENT CDCl3
NS 24
DS 16
SWH 2997.602 Hz
FIDRES 0.731836 Hz
AQ 0.6832628 sec
RG 16384
DW 166.800 usec
DE 6.50 usec
TE 295.3 K
CNST2 145.0000000
CNST13 8.0000000
D0 0.00000300 sec
D1 1.20918405 sec
D2 0.00344828 sec
D6 0.06250000 sec
D16 0.00020000 sec
IN0 0.00002235 sec

======== CHANNEL f1 ========
NUC1 1H
P1 12.00 usec
P2 24.00 usec
PL1 1.10 dB
SFO1 400.1315246 MHz

======== CHANNEL f2 ========
NUC2 13C
P3 8.00 usec
PL2 2.50 dB
SFO2 100.6228138 MHz

====== GRADIENT CHANNEL =====
GPNAM1 SINE.100
GPNAM2 SINE.100
GPNAM3 SINE.100
GPZ1 50.00 %
GPZ2 30.00 %
GPZ3 40.10 %
P16 1000.00 usec
ND0 2
TD 320
SFO1 100.6228 MHz
FIDRES 69.836967 Hz
SW 222.095 ppm
FnMODE QF
SI 2048
SF 400.1300354 MHz
WDW SINE
SSB 0
LB 0.00 Hz
GB 0
PC 1.40
SI 1024
MC2 QF
SF 100.6127690 MHz
WDW SINE
SSB 0
LB 0.00 Hz
GB 0

HMBCGP CDCl3 {C:\Bruker\TOPSPIN} dcro 29

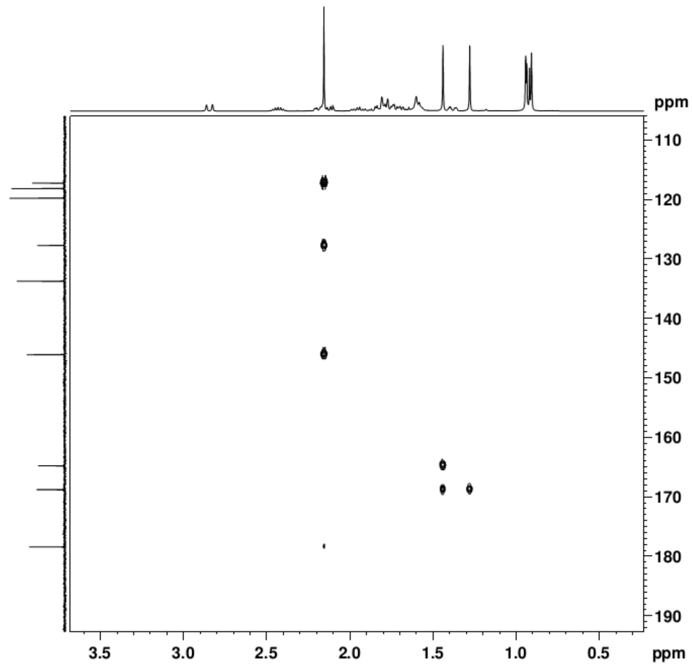


```
NAME                may
EXPNO               500
PROCNO                1
Date_          20121014
Time              13.14
INSTRUM           spect
PROBHD   5 mm Dual 13C/
PULPROG     hmbcgplpndqf
TD                 4096
SOLVENT           CDCl3
NS                   24
DS                   16
SWH            2997.602 Hz
FIDRES         0.731836 Hz
AQ            0.6832628 sec
RG                16384
DW              166.800 usec
DE                 6.50 usec
TE                295.3 K
CNST2       145.0000000
CNST13        8.0000000
D0           0.00000300 sec
D1           1.20918405 sec
D2           0.00344828 sec
D6           0.06250000 sec
D16          0.00020000 sec
IN0          0.00002235 sec

======== CHANNEL f1 ========
NUC1                 1H
P1                12.00 usec
P2                24.00 usec
PL1                1.10 dB
SFO1        400.1315246 MHz

======== CHANNEL f2 ========
NUC2                13C
P3                 8.00 usec
PL2                2.50 dB
SFO2        100.6228138 MHz

====== GRADIENT CHANNEL =====
GPNAM1         SINE.100
GPNAM2         SINE.100
GPNAM3         SINE.100
GPZ1              50.00 %
GPZ2              30.00 %
GPZ3              40.10 %
P16             1000.00 usec
ND0                   2
TD                  320
SFO1           100.6228 MHz
FIDRES        69.836967 Hz
SW              222.095 ppm
FnMODE               QF
SI                 2048
SF          400.1300354 MHz
WDW                SINE
SSB                   0
LB                 0.00 Hz
GB                    0
PC                 1.40
SI                 1024
MC2                  QF
SF          100.6127690 MHz
WDW                SINE
SSB                   0
LB                 0.00 Hz
GB                    0
```

HMBCGP CDCl3 {C:\Bruker\TOPSPIN} dcro 29

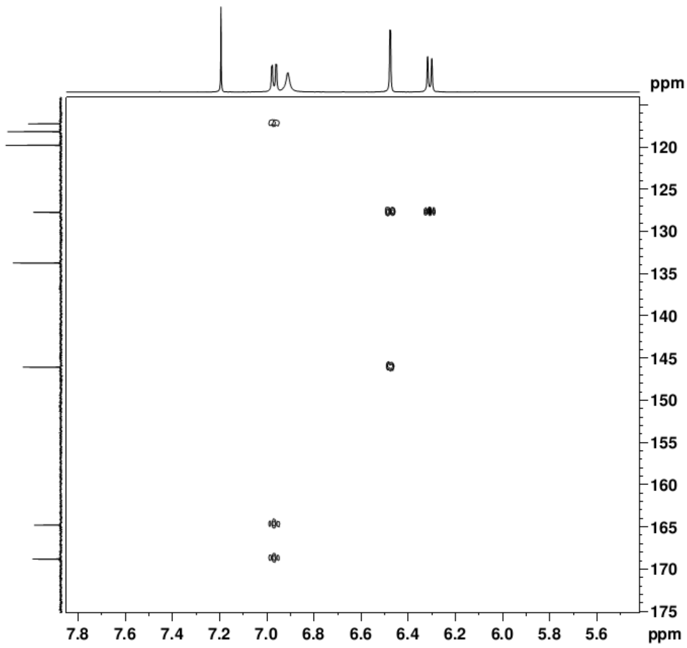

NAME may
EXPNO 500
PROCNO 1
Date_ 20121014
Time 13.14
INSTRUM spect
PROBHD 5 mm Dual 13C/
PULPROG hmbcgplpndqf
TD 4096
SOLVENT CDCl3
NS 24
DS 16
SWH 2997.602 Hz
FIDRES 0.731836 Hz
AQ 0.6832628 sec
RG 16384
DW 166.800 usec
DE 6.50 usec
TE 295.3 K
CNST2 145.0000000
CNST13 8.0000000
D0 0.00000300 sec
D1 1.20918405 sec
D2 0.00344828 sec
D6 0.06250000 sec
D16 0.00020000 sec
IN0 0.00002235 sec
======== CHANNEL f1 ========
NUC1 1H
P1 12.00 usec
P2 24.00 usec
PL1 1.10 dB
SFO1 400.1315246 MHz
======== CHANNEL f2 ========
NUC2 13C
P3 8.00 usec
PL2 2.50 dB
SFO2 100.6228138 MHz
====== GRADIENT CHANNEL =====
GPNAM1 SINE.100
GPNAM2 SINE.100
GPNAM3 SINE.100
GPZ1 50.00 %
GPZ2 30.00 %
GPZ3 40.10 %
P16 1000.00 usec
ND0 2
TD 320
SFO1 100.6228 MHz
FIDRES 69.836967 Hz
SW 222.095 ppm
FnMODE QF
SI 2048
SF 400.1300354 MHz
WDW SINE
SSB 0
LB 0.00 Hz
GB 0
PC 1.40
SI 1024
MC2 QF
SF 100.6127690 MHz
WDW SINE
SSB 0
LB 0.00 Hz
GB 0

HMBCGP CDCl3 {C:\Bruker\TOPSPIN} dcro 29

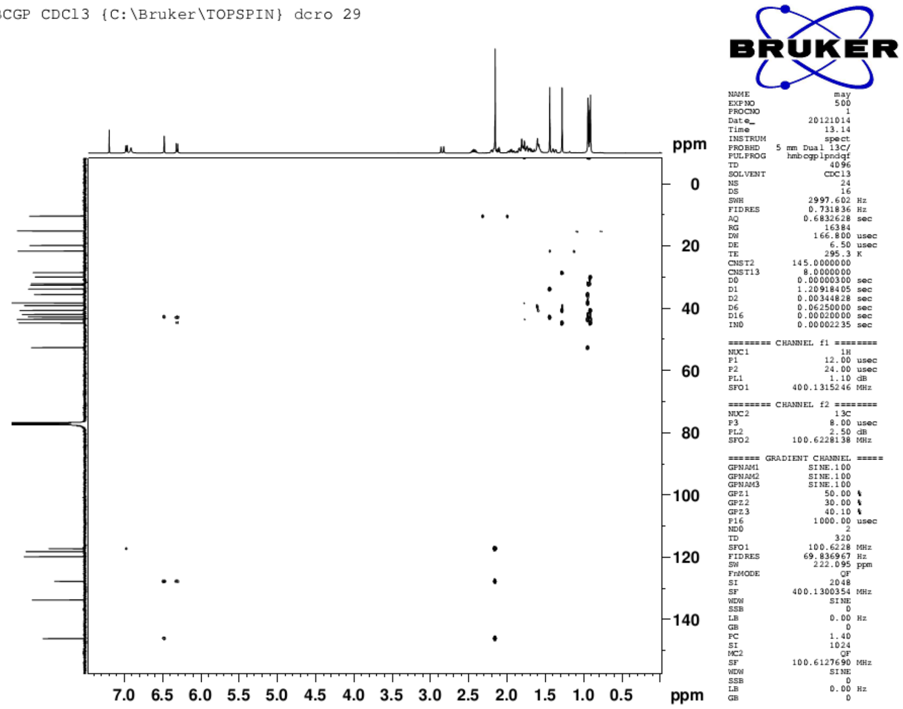

HSQCETGP CDCl3 {C:\Bruker\TOPSPIN} dcro 29

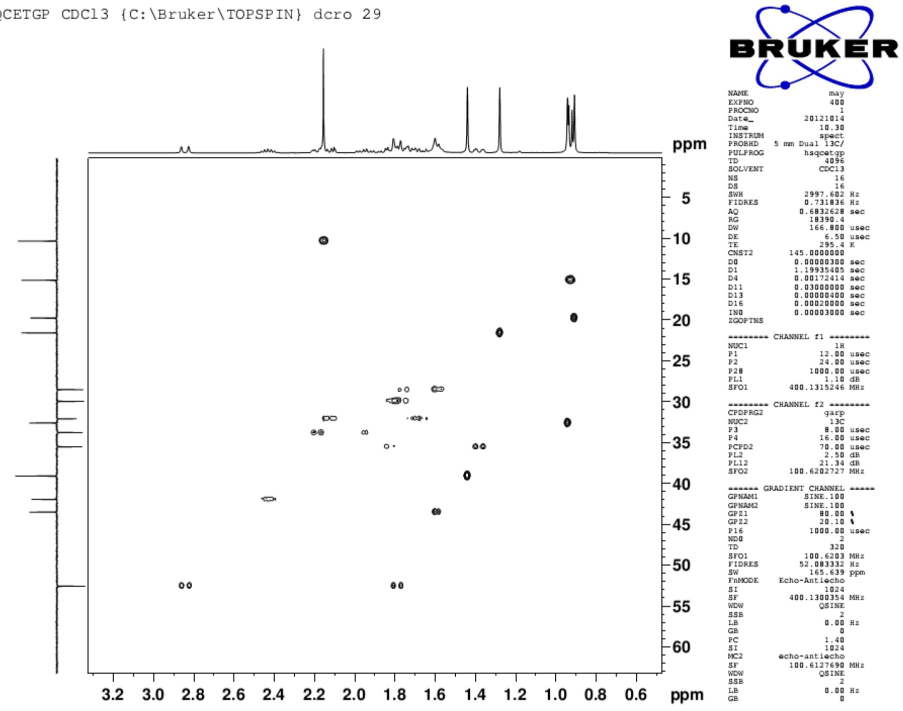

HSQCETGP CDCl3 {C:\Bruker\TOPSPIN} dcro 29

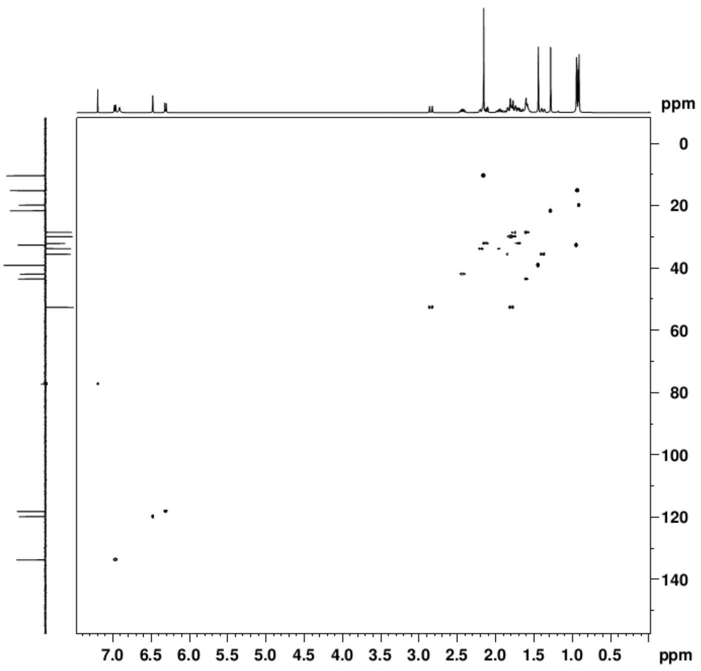


```
NAME                may
EXPNO               400
PROCNO                1
Date_          20121014
Time              10.30
INSTRUM           spect
PROBHD   5 mm Dual 13C/
PULPROG        hsqcetgp
TD                 4096
SOLVENT           CDCl3
NS                   16
DS                   16
SWH            2997.602 Hz
FIDRES         0.731836 Hz
AQ            0.6832628 sec
RG              18390.4
DW              166.800 usec
DE                 6.50 usec
TE                295.4 K
CNST2       145.0000000
D0           0.00000300 sec
D1           1.19935405 sec
D4           0.00172414 sec
D11          0.03000000 sec
D13          0.00000400 sec
D16          0.00020000 sec
IN0          0.00003000 sec
ZGOPTNS

======== CHANNEL f1 ========
NUC1                 1H
P1                12.00 usec
P2                24.00 usec
P28             1000.00 usec
PL1                1.10 dB
SFO1        400.1315246 MHz

======== CHANNEL f2 ========
CPDPRG2            garp
NUC2                13C
P3                 8.00 usec
P4                16.00 usec
PCPD2             70.00 usec
PL2                2.50 dB
PL12              21.34 dB
SFO2        100.6202727 MHz

====== GRADIENT CHANNEL =====
GPNAM1         SINE.100
GPNAM2         SINE.100
GPZ1              80.00 %
GPZ2              20.10 %
P16             1000.00 usec
ND0                   2
TD                  320
SFO1           100.6203 MHz
FIDRES        52.083332 Hz
SW              165.639 ppm
FnMODE    Echo-Antiecho
SI                 1024
SF          400.1300354 MHz
WDW               QSINE
SSB                   2
LB                 0.00 Hz
GB                    0
PC                 1.40
SI                 1024
MC2       echo-antiecho
SF          100.6127690 MHz
WDW               QSINE
SSB                   2
LB                 0.00 Hz
GB                    0
```
